# Carotid Artery Doppler: A Possible Non-invasive Diagnostic Approach to Assessing the Severity of Coronary Artery Disease

**DOI:** 10.7759/cureus.62886

**Published:** 2024-06-22

**Authors:** Ritika Agarwal, Jahnavi Gadupati, Sampangi S Ramaiah, Varsha G Babu, Aditi Jain, V. S. Prakash

**Affiliations:** 1 Radiology, M. S. Ramaiah Medical College, Bengaluru, IND; 2 Cardiology, M. S. Ramaiah Medical College, Bengaluru, IND

**Keywords:** significant stenosis, multi-vessel disease, carotid artery doppler, coronary artery disease, gensini score

## Abstract

Background and objectives: Carotid artery Doppler ultrasound is being explored for its potential as a non-invasive tool for evaluating coronary artery disease (CAD) severity and cardiovascular risk. This study aimed to investigate the association between carotid Doppler parameters and CAD severity, as determined by the Gensini score (GS) and multi-vessel disease presence.

Methods: Ninety patients undergoing coronary angiography (CAG) and carotid ultrasound were retrospectively analysed. Carotid Doppler parameters and CAD severity were assessed through bivariate and multivariable analyses.

Results: Among the patients studied, 80% were male, with a mean age of 65.24 years (±9.91). Triple vessel disease (68.9%) and dominant vessel disease (73.3%) were prevalent coronary findings, with a mean GS of 139.2 (±76.6). Increased intima-media thickness (IMT) in the common carotid artery (CCA) and internal carotid artery (ICA) showed significant associations (CCA IMT: OR = 0.312, p = 0.037; ICA IMT: OR = 0.354, p = 0.017) with high GS (>125) and multi-vessel disease. Significant carotid stenosis (>50% diameter stenosis) emerged as an independent predictor of CAD severity. Fibrocalcific plaques, detected in 62.2% of cases, correlated significantly with elevated GS. Plaque burden, especially plaques in >3 locations, indicated a higher likelihood of triple vessel disease and a higher GS.

Conclusion: Carotid Doppler parameters, particularly IMT and significant stenosis, are robust predictors of CAD severity, including high GS and multi-vessel disease. Integrating carotid artery assessment into clinical protocols can aid in timely interventions and preventive strategies for CAD management.

## Introduction

Atherosclerosis stands as the principal contributor to the majority of cases involving coronary artery disease (CAD). Given its systemic nature, a comprehensive assessment across multiple vascular sites may offer deeper insights into the overall burden and risk of subclinical atherosclerosis [1]. Detecting subclinical illness before symptoms manifest is paramount for public health initiatives. Consequently, there exists a critical need to diagnose subclinical atherosclerosis to enhance CAD risk stratification in asymptomatic individuals, prioritizing them for primary prevention interventions [1].

Presently, coronary angiography (CAG) serves as the primary clinical method for diagnosing coronary atherosclerosis. However, its invasive nature, high cost, and technical complexity pose significant challenges [2]. Therefore, there is a concerted effort to predict CAD severity and complexity using noninvasive methods to identify high-risk patients for cardiovascular events. Notably, the structural and mechanistic parallels between carotid and coronary arteries in atherosclerosis pathogenesis have spurred interest in carotid artery assessment as a surrogate [3,4]. Carotid ultrasound emerges as a promising alternative, offering simplicity in operation, reliable data, safety, repeatability, and widespread clinical application [2,5,6].

Our objective is to evaluate the efficacy of carotid artery Doppler ultrasound for screening atherosclerosis and assessing coronary artery conditions in individuals who have undergone invasive CAG for suspected CAD. We aim to explore the relationship between various carotid Doppler parameters (including carotid intima-media thickness (IMT), plaque number and characteristics, peak systolic velocity (PSV), pulsatility index (PI), and resistive index (RI)) and dominant main CAD, three-vessel CAD, and Gensini score (GS). This investigation seeks to harness the potential of carotid artery Doppler in predicting CAD, thereby facilitating timely clinical detection and prevention efforts against CAD.

## Materials and methods

Study population

In this retrospective study conducted at Ramaiah Memorial Hospital, Bangalore, from January to June 2023, a cohort of 90 patients presenting with atypical chest pain and undergoing both CAG and carotid ultrasound during the same admission was retrospectively recruited for analysis. The study examined various clinical and demographic characteristics of the patients, including age, gender, presence of comorbidities such as diabetes mellitus, hypertension, hyperlipidemia, and history of smoking.

The inclusion criteria comprised patients with any form of chest pain indicative of ischemic heart disease necessitating evaluation through invasive CAG. We excluded individuals with a documented history of CAD (such as myocardial infarction or prior revascularization procedures), carotid interventions (such as endarterectomy or stenting), previous strokes, or patients deemed clinically unstable to undergo the duration of CAG [7].

Hypertension was defined as a blood pressure measurement of ≥140/90 mmHg or the use of antihypertensive medication; hyperlipidemia as a fasting low-density lipoprotein concentration exceeding 130 mg/dL or the use of antihyperlipidemic medication; and diabetes mellitus as a fasting plasma glucose concentration of ≥126 mg/dL or the use of antidiabetic medication [7]. The study received approval from the ethics committee at Ramaiah Medical College (Reg No. ECR/215/Inst/KA/2013/RR-22).

Assessment of coronary angiogram and Gensini score

A coronary angiogram was obtained either through the radial or femoral route. Experienced independent cardiologist observers, blinded to both the patient's clinical characteristics and carotid Doppler US findings, assessed the CAG.

Significant and severe CAD were assumed to have a lumen diameter stenosis of ≥50% and >70%, respectively, in any of the major epicardial coronary arteries, including the left main coronary artery, left anterior descending artery, left circumflex artery, and right coronary artery. CAD is categorized as a one-vessel disease (1-VD; disease in 1 vessel), a two-vessel disease (2-VD; disease in 2 vessels), a three-vessel disease (3-VD; disease in 3 vessels or left main trunk disease with right coronary artery stenosis), and a dominant vessel disease [7]. Coronary arterial dominance is determined by the vessel that gives rise to the posterior descending artery.

**Figure 1:**
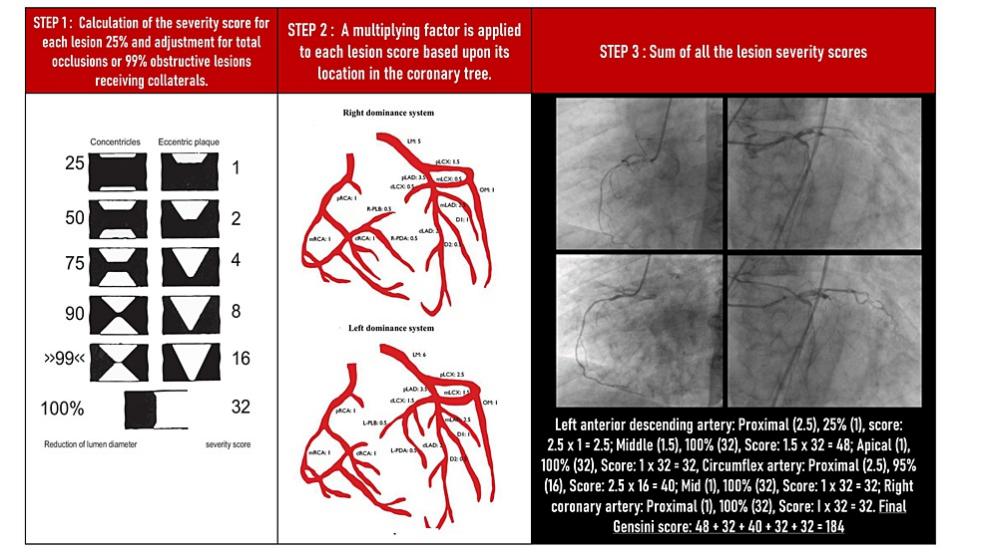
Calculation of Gensini score pRCA: proximal right coronary artery; mRCA: mid right coronary artery; dRCA: distal right coronary artery; PDA: posterior descending artery; PLB: posterolateral branch; pLAD: proximal left anterior descending artery; mLAD: mid left anterior descending artery; dLAD: distal left anterior descending artery; pLCx: proximal left circumflex; mLCx: mid left circumflex; dLCx: distal left circumflex; OM: obtuse marginal; D1: diagonal branch 1; D2: diagonal branch 2

GS was calculated by giving each coronary stenosis a severity value and then summating individual coronary segment scores [8] (Figure 1). The patients were classified into three groups according to the tertile of GS: group 1 (GS ≤125); group 2 (score between 126 and 200); and group 3 (score > 200). 

Carotid artery Doppler ultrasound

Carotid artery Doppler ultrasound examinations were conducted by skilled radiologists utilizing B-mode imaging and duplex ultrasonography. Evaluations encompassed the right and left CCAs as well as the ICAs. Patients were instructed to abstain from alcohol and nicotine for a period of 12 hours preceding the examination.

**Figure 2:**
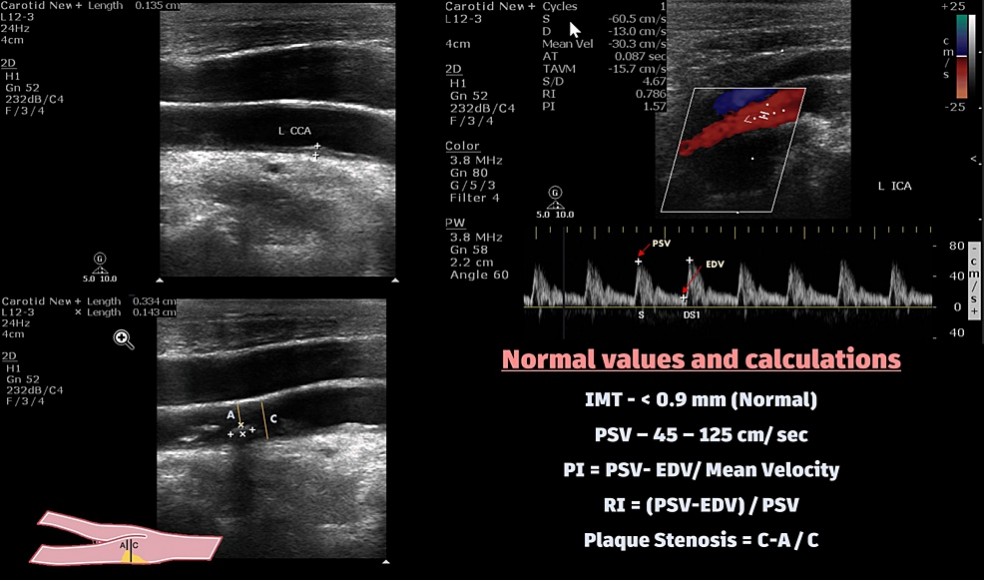
Assessment with carotid artery Doppler parameters and normal values IMT: intima-media thickness; PSV: peak systolic velocity; EDV: end diastolic volume; PI: Pulsatility Index; RI: Resistivity Index

In our study, we assessed various carotid Doppler parameters aimed at identifying potential indicators of cardiovascular risk. IMT measurements were acquired from grayscale images, delineating the distance between the intima-lumen interface (echogenic layer) and the media-adventitia border (echo-poor layer) along the far wall of both the CCA and ICA. Additionally, we analysed PSV, PI, and RI to gain insights into vascular resistance and function (Figure 2). Furthermore, we evaluated the presence and characteristics of plaques, including their size, composition, and distribution along the carotid arteries.

The assessment of stenosis severity was based on two key parameters: the maximum percentage of diameter reduction recorded via B-mode ultrasonography and the PSV measured within the carotid arteries. Patients were stratified into categories of no stenosis, mild stenosis, moderate stenosis, and severe stenosis according to these parameters [9]. Specifically:

- No diameter stenosis or ICA PSV <125 cm/sec was classified as no stenosis.

- Diameter reduction <50% with ICA PSV <125 cm/sec was categorized as mild stenosis.

- Diameter stenosis of 50-69% with PSV of 125-230 cm/sec was considered moderate stenosis.

- Near-total occlusion, total occlusion, or 70-99% occlusion with either PSV >230 cm/sec or undetectable flow were classified as severe stenosis.

- Moderate and Severe stenosis was considered significant stenosis in our study.

Statistical analysis

All the data were analysed using IBM SPSS version 22.0 (IBM Corp., Armonk, NY). Continuous and categorical variables were represented as mean ± standard deviation and as frequency (percentage), respectively. The association between various carotid Doppler parameters and the severity of CAD was determined using bivariate analysis utilizing the chi-square test.

Multivariable logistic regression was performed to determine parameters predicting the severity of CAD by including parameters showing P-values ≤ 0.05 at the univariable analysis. The odds ratio (OR) and 95% confidence intervals (CIs) were calculated. The sensitivity, specificity, and positive predictive value (PPV) of the carotid Doppler parameters were also calculated. Statistical significance was considered for a P-value < 0.05.

## Results

The demographic profile of the patients studied is shown in Table 1. Among the 90 study patients, 72 (80%) were males. The mean age of the study population was 65.2 ± 9.92 years. Analysis of coronary parameters revealed varied degrees of disease severity, with the majority presenting with triple vessel disease (68.9%) and dominant vessel involvement (73.3%). The mean GS, indicative of CAD severity, was 139.2 ± 76.6, distributed across categories including ≤125 (42.2%), 126-200 (42.2%), and >200 (15.6%). Carotid assessments highlighted significant IMT and peak systolic velocity measurements across both CCAs and ICAs.

**Table 1:** Baseline details and findings on coronary angiogram and carotid Doppler ultrasound of the study patients R-CCA: right common carotid artery; L-CCA: left common carotid artery; R-ICA: right internal carotid artery; L-ICA: left internal carotid artery; IMT: intima-media thickness; PSV: peak systolic velocity

Characteristics	n%/mean ± standard deviation
Age (years)	65.24 ± 9.91
Male	72 (80)
Risk factors	
Hypertension	46 (51.1)
Diabetes	28 (31.1)
Dyslipidemia	19 (21.1)
Smoking	21 (23.3)
Alcoholism	15 (16.6)
Coronary parameters	
No disease	4 (4.4)
Single vessel disease	4 (4.4)
Double vessel disease	20 (22.2)
Triple vessel disease	62 (68.9)
Dominant vessel disease	66 (73.3)
Gensini score	139.2 ± 76.6
Gensini score (≤125)	38 (42.2)
Gensini score (126-200)	38 (42.2)
Gensini score (>200)	14 (15.6)
Carotid Parameters	
R-CCA IMT	1.17 ± 0.35
R-ICA IMT	1.04 ± 0.28
L-CCA IMT	1.14 ± 0.32
L-ICA IMT	1.05 ± 0.26
R-CCA PSV	81.6 ± 26.6
R-ICA PSV	75.1 ± 44.4
L-CCA PSV	86.8 ± 28.2
L-ICA PSV	76.3 ± 32.6
Plaques in 1 location	10 (11.1)
Plaques in 2 locations	26 (28.9)
Plaques in 3 locations	8 (8.9)
Plaques in 4 locations	20 (22.2)
Significant stenosis in R-CCA	21 (23.3)
Significant stenosis in R-ICA	35 (38.8)
Significant stenosis in L-CCA	20 (22.2)
Significant stenosis in L-ICA	39 (43.3)

Individuals with CAD showed a distinct pattern of carotid IMT, with IMT exceeding 1.1 mm and 1.3 mm in the ICA and CCA being significantly associated with the presence of dominant vessel disease, GS (>125), and the prevalence of triple vessel disease. Moreover, the number of plaque locations across the carotid arteries was positively associated with coronary vessel disease severity, with a higher number of plaque locations indicating a greater likelihood of triple vessel disease and higher GS. The composition of carotid plaques also displayed significant associations, with fibrocalcific plaques showing the strongest correlation with coronary vessel disease severity (Table 2).

**Table 2:** Percentages of coronary vessel disease and Gensini scores according to the various parameters studied in the carotid artery Doppler IMT: intima-media thickness; CCA: common carotid artery; ICA: internal carotid artery, R-CCA: right common carotid artery, L-CCA: left common carotid artery; R-ICA: right internal carotid artery; L-ICA: left internal carotid artery

	Coronary vessel disease (% of total)						Gensini score (% of total)			
	No disease (4/90)	Dominant (66/90)	Single (4/90)	Double (20/90)	Triple (62/90)	P-value	≤125	126-200	>200	P-value
IMT >1.1 mm in CCA	0	57.8	2.2	4.4	57.8	<0.001	17.8	31.1	15.6	.013
IMT >1.3 mm in CCA	0	37.8	0	4.4	35.6	0.009	8.9	17.8	13.3	0.011
IMT >1.1 mm in ICA	0	53.3	2.2	6.7	51.1	0.024	15.6	28.9	15.6	0.009
Significant stenosis in ICA or CCA	0	55.6	0	4.4	51.1	0.002	4.4	35.6	15.6	<0.001
Number of locations with plaques (locations: R-CCA, R-ICA, L-CCA and L-ICA)										
0	4.4	6.7	4.4	15.6	4.4	<0.001	22.2	6.7	0	0.004
1	0	11.1	0	0	11.1	<0.001	2.2	8.9	0	0.004
2	0	26.7	0	2.2	26.7	<0.001	13.3	11.1	4.4	0.004
3	0	6.7	0	2.2	6.7	<0.001	4.4	4.4	0	0.004
4	0	22.2	0	2.2	20	<0.001	0	26.3	71.4	0.004
Fibrofatty plaques	0	25.6	0	4.4	22.2	.536	6.7	11.1	8.9	0.107
Fibrocalcific plaques	0	62.2	0	6.7	60	<0.001	17.8	33.3	15.6	0.007

Increased CCA and ICA IMT are associated with increased odds of having a dominant vessel and triple vessel disease. Multivariate analysis of the predictor variables (P < 0.01) in a regression model showed that significant stenosis in any carotid vessel was the only independent predictor of high GS, dominant vessel disease, and triple vessel disease in the study population (Table 3).

**Table 3:** Regression model for high Gensini score (>125), dominant vessel disease, double and triple vessel disease, and carotid artery Doppler parameters IMT: intima-media thickness; CCA: common carotid artery; PSV: peak systolic velocity; PI: Pulsatility Index; RI: Resistivity Index; ICA: internal carotid artery

	OR (univariable)	OR (multivariable)
Dependant: high Gensini score (>125)		
CCA IMT	0.312 (0.176–2.154, p=0.037)	0.105 (0.101–0.811, p=0.764)
CCA PSV	0.194 (0.112–1.300, p=0.201)	
CCA PI	0.074 (0.150–0.484, p=0.631)	
CCA RI	0.201 (0.148–1.343, p=0.186)	
ICA IMT	0.354 (0.178–2.482, p=0.017)	0.365 (0.192–1.623, p=0.560)
ICA PSV	0.169 (0.119–1.127, p=0.266)	
ICA PI	0.304 (0.125–2.096, p=0.042)	0.119 (0.109 -0.346, p=0.297)
ICA RI	0.140 (0.144–0.929, p=0.0358)	
Significant stenosis in any carotid vessel	0.775 (0.095–8.033, p<0.001)	0.714 (0.504–0.925, p≤0.001)
Dependant: dominant vessel disease		
CCA IMT	0.358 (0.121–2.511, p=0.016)	0.040 (0.011–0.747, p=0.911)
CCA PSV	0.053 (0.131–0.348, p=0.730)	
CCA PI	0.085 (0.100–0.559, p=0.579)	
CCA RI	0.026 (0.140–0.169, p=0.867)	
ICA IMT	0.394 (0.102–2.813, p=0.007)	0.665 (0.596–1.945, p=0.292)
ICA PSV	0.249 (0.89–1.687, p=0.99)	
ICA PI	0.119 (0.149–0.783, p=0.438)	
ICA RI	0.175 (0.154–1.166, p=0.250)	
Significant stenosis in any carotid vessel	0.674 (0.087–5.986, p<0.001)	0.542 (0.331–0.752, p<0.001)
Dependant: double vessel disease		
CCA IMT	0.417 (0.112–3.006, p=0.004)	0.188 (0.128–0.479, p=0.566)
CCA PSV	0.152 (0.146–1.006, p=0.320)	
CCA PI	0.121 (0.091–0.800, p=0.428)	
CCA RI	0.023 (0.127–0.150, p=0.82)	
ICA IMT	0.298 (0.121–2.04, p=0.47)	0.153 (0.059–1.018, p=0.415)
ICA PSV	0.033 (0.023–0.219, p=0.828)	
ICA PI	0.060 (0.030–0.391, p=0.698)	
ICA RI	0.089 (0.042–0.132, p=0.895)	
Significant stenosis in any carotid vessel	0.382 (0.131–2.714, p =0.010)	0.254 (0.124–0.517, p=0.059)
Dependant: triple vessel disease		
CCA IMT	0.380 (0.134–2.769, p=0.008)	0.167 (0.112–1.012, p=0.688)
CCA PSV	0.124 (0.100–0.818, p=0.418)	
CCA PI	0.153 (0.150–1.016, p=0.315)	
CCA RI	0.165 (0.144–1.094, p=0.280)	
ICA IMT	0.445 (0.094–3.259, p=0.002)	0.716 (0.453–2.20, p=0.338)
ICA PSV	0.028 (0.128–0.187, p=0.853)	
ICA PI	0.088 (0.128–0.579, p=0.566)	
ICA RI	0.193 (0.151–1.287, p=0.205)	
Significant stenosis in any carotid vessel	0.558 (0.119–4.411, p<0.001)	0.432 (0.182–0.681, p=0.01)

For high GS (>125), triple vessel disease and dominant vessel disease parameters such as an IMT exceeding 1.3 mm in the CCA and 1.1 mm in the ICA, significant stenosis in either the ICA or CCA demonstrated high sensitivities from 48.6% to 88.5%, high specificities from 63.2% to 90%, and PPVs from 74.1% to 96%. These results underscore the potential of carotid Doppler parameters, particularly IMT measurements and the presence of significant stenosis, as valuable indicators for assessing CAD severity (Table 4).

**Table 4:** Performance of carotid Doppler parameters for coronary artery disease severity IMT: intima-media thickness; CCA: common carotid artery; PSV: peak systolic velocity; ICA: internal carotid artery

	Sensitivity %	Specificity %	PPV %	p-value
High Gensini score (>125)				
IMT >1.3 mm in CCA	53.8	78.9	77.8	<0.05
IMT >1.1 mm in ICA	76.9	63.2	74.1	0.007
PSV >125 in CCA	23.1	100	100	0.024
Plaques in >3 locations	46.2	89.5	85.7	0.011
Significant stenosis in ICA or CCA	88.5	89.5	92	<0.001
Severe stenosis in a dominant vessel (>70%)				
IMT >1.3 mm in CCA	48.6	90	94.4	<0.05
IMT >1.1 mm in ICA	71.4	80	92.6	0.003
PSV >125 in CCA	17.1	100	100	0.160
Plaques in >3 locations	37.1	90	92.9	0.102
Significant stenosis in ICA or CCA	68.6	90	96	0.001
Triple vessel disease				
IMT >1.3 mm in CCA	51.6	85.7	88.9	<0.05
IMT >1.1 mm in ICA	74.2	71.4	85.2	0.004
PSV >125 in CCA	16.1	92.9	83.3	0.412
Plaques in >3 locations	38.7	85.7	85.7	0.101
Significant stenosis in ICA or CCA	74.2	85.7	92	<0.001

## Discussion

The findings from this study underscore the potential of carotid artery Doppler parameters, including IMT measurements, stenosis severity, plaque distribution, and composition, as valuable indicators for assessing CAD severity and risk stratification.

Several studies have demonstrated the association between increased IMT in the carotid arteries and the presence and severity of CAD. For instance, a meta-analysis by Lorenz et al. [10] found a positive correlation between carotid IMT and the prevalence and severity of CAD, suggesting IMT as a marker for subclinical atherosclerosis and increased cardiovascular risk. Moreover, the presence of significant stenosis in the carotid arteries has been consistently linked to CAD severity. Studies by Pathakota et al. [11], O'Leary et al. [12], and Saedi et al. [13] showed that carotid artery stenosis, assessed by Doppler ultrasound, was independently associated with the presence of CAD, highlighting the prognostic value of carotid stenosis in predicting CAD severity and cardiovascular events. 

In the studies by Kasliwal et al. [14] and Naqvi and Lee [15], a significant association was found between elevated carotid IMT and the presence of multi-vessel CAD, highlighting the utility of IMT measurement as a marker of CAD severity. Consistent with these findings, our study observed a positive correlation between increased carotid IMT values and CAD severity, further supporting the prognostic value of IMT assessment in CAD risk prediction.

Our findings also align with a recent study by Pakizer et al. [16], which reported that elevated PSV in the carotid arteries was associated with an increased risk of CAD events. This suggests that PSV measurements may serve as a valuable prognostic marker for CAD progression and adverse cardiovascular outcomes. PSV >125 in the CCA exhibited excellent specificity (100%) but limited sensitivity (23.1%) for high GS, indicating its potential as a confirmatory test rather than a screening tool in our study.

In the context of severe stenosis in the dominant vessel, carotid IMT measurements >1.3 mm in the CCA and >1.1 mm in the ICA exhibit moderate to high sensitivity and specificity, with IMT >1.3 mm in the CCA showing promising performance in terms of sensitivity (48.6%) and specificity (90%). These results corroborate the notion of the study by Sibal et al. [17] that carotid IMT can serve as a surrogate marker for CAD severity in high-risk populations.

In the context of GS, the univariable analysis showed significant associations between PI of the ICA and high GS (>125) (OR: 0.304, p = 0.042). This suggests that elevated PI values may serve as a predictor of advanced CAD, reflecting alterations in carotid artery hemodynamics associated with atherosclerosis progression. Furthermore, the multivariable analysis revealed that the RI of the ICA remained independently associated with a high GS (>125) after adjusting for potential confounders. The RI of ICA demonstrated a significant association with high GS (>125) (OR: 0.140, p = 0.0358) in the univariable analysis, suggesting that increased ICA RI values may indicate more severe CAD. This is similar to the study by Yang Li et al. [18], who investigated the relationship between carotid artery Doppler ultrasonography pulsatility index and CAD severity. Their results demonstrated a positive correlation between pulsatility index values and the severity of CAD, with higher pulsatility index values associated with more extensive CAD. Hitomi et al. [19] explored the association of carotid artery Doppler ultrasonography parameters with CAD severity and followed up for 5.9 ± 3.2 years for the development of adverse cardiac events. Their study revealed that carotid artery PIs were independent predictive factors for further cardiovascular events.

Wang and He [20] and Seo et al. [21] further explored the association between carotid artery stenosis and CAD severity in a diverse patient population. Their study revealed a dose-response relationship, wherein increasing degrees of carotid stenosis were linked to higher GS and a greater likelihood of multi-vessel CAD. These outcomes, akin to our own, underscore the additional predictive capacity of carotid stenosis in stratifying CAD risk, suggesting its utility as a non-invasive marker for identifying individuals at heightened risk of adverse cardiovascular events.

Ioannis Kallikazaros et al. [22] investigated the predictive value of carotid artery stenosis for CAD severity and clinical outcomes in a prospective study. Their findings echoed those of previous research, demonstrating a strong association between significant carotid stenosis and severe CAD. Importantly, they observed that patients with significant carotid stenosis were more prone to experiencing cardiovascular events, emphasizing the clinical relevance of assessing carotid stenosis in CAD risk assessment and management. Our study builds upon these findings by providing additional insights into the relationship between significant carotid stenosis and CAD severity.

Plaque burden, particularly the presence of plaques in >3 locations, demonstrates moderate sensitivity (46.2%) and high specificity (89.5%), suggesting its role as a complementary marker for CAD severity assessment. A study by Tada et al. [23] demonstrated that carotid plaque burden was independently associated with CAD severity and adverse cardiovascular outcomes, further emphasizing the prognostic value of carotid plaque assessment in CAD risk stratification.

The presence of fibrocalcific plaques has shown significant associations with coronary vessel disease severity and higher GS in our study. This suggests that the composition of carotid plaques, particularly the presence of fibrocalcific plaques, may provide valuable insights into the extent and severity of CAD. Similarly, in the studies by Spagnoli et al. [24] and Mathiesen et al. [25], the presence of fibrocalcific plaques in the carotid arteries was significantly associated with CAD severity, highlighting the importance of plaque characterization in risk prediction models.

Honda et al. [26] found that the presence of echolucent plaques, indicative of lipid-rich and vulnerable plaques, was significantly associated with the severity of CAD and increased cardiovascular events. Our study corroborates these findings by demonstrating a positive correlation between the composition of carotid plaques.

Overall, the findings from our study align with existing literature, further supporting the utility of carotid artery Doppler parameters, including IMT measurements, stenosis severity, and plaque characteristics, as valuable tools for assessing CAD severity and cardiovascular risk.

Further validation of the results is necessary for a larger and more diverse population across multiple centers. Our study did not utilize intravascular ultrasound, which is widely regarded as the gold standard for plaque characterization [27]. The study population consisted primarily of symptomatic patients referred for CAG, potentially introducing a bias towards a higher prevalence of obstructive CAD. Future research should encompass larger cohorts with varied patient demographics to ensure the generalizability of our findings.

## Conclusions

In summary, our research highlights the importance of using carotid artery Doppler ultrasound to evaluate CAD severity and cardiovascular risk. By examining factors like IMT, plaque features, and hemodynamic markers, we uncovered significant links to CAD severity and multi-vessel disease. Particularly, identifying significant carotid stenosis proved highly predictive of CAD severity, with plaque analysis offering additional valuable information. Our study emphasizes the need to incorporate carotid artery assessment into clinical protocols for better CAD risk assessment. By leveraging the non-invasive nature and diagnostic precision of carotid Doppler ultrasound, clinicians can proactively identify individuals at heightened risk of adverse cardiovascular events, thus facilitating timely interventions and preventive strategies. However, further investigations are needed to confirm these results and delve into personalized CAD management strategies.
